# Ethical Concerns Regarding High-Dose Enoxaparin for Venous Thromboembolism Prevention in Plastic Surgery Patients

**DOI:** 10.1097/GOX.0000000000002485

**Published:** 2019-08-30

**Authors:** Eric Swanson

**Affiliations:** From the Swanson Center, Leawood, Kans.

## Sir:

In a study in progress, plastic surgery patients are randomized into 2 groups treated with enoxaparin 40 mg bid or 0.5 mg/kg bid (35 mg bid for a 70-kg patient).^1^ Although anti-Factor Xa levels reflect enoxaparin activity,^1^ these levels do not directly measure the anticoagulant effect.^2^ The authors depend on clinical signs to diagnose venous thromboembolism (VTE),^1^ which are unreliable.^3^ Although the study began in July, 2017, no data are reported.^1^

The U.S. Food and Drug Administration approved enoxaparin for VTE prophylaxis only in high-risk general surgery and joint replacement patients.^4^ The recommended daily dose is 30 or 40 mg.^5^ Plastic surgeons should be aware that prescribing enoxaparin to prevent VTEs in plastic surgery patients is off-label. A dosing schedule of 40 mg bid, double the usual prophylactic dose, produces a 6.8% rate of clinically relevant bleeding.^6^

Thirty percent of patients receiving 40 mg of enoxaparin twice daily are over-anticoagulated, as indicated by anti-Factor Xa levels, and these patients are likely to suffer more bleeding, returns to the operating room, blood transfusions, and death.^7^ The authors seek to determine whether a variable weight-based dosing regimen reduces the frequency of overdoses.^1^ However, the study design calls for half of the patients to receive a fixed 40 mg bid enoxaparin dose regardless of body weight, compromising the ethical requirement of equipoise.^8^ A compensatory benefit is unclear.^3,9^ Clearly, patients and institutional review boards need to be informed of the unapproved regulatory status and additional bleeding risk. The study is underpowered to detect a difference in VTEs or bleeding,^1^ but this limitation may be overshadowed by ethical considerations. Perhaps fewer patients will receive overdoses with weight-based dosing,^1^ but even one overdose is unacceptable.

A short in-hospital course (eg, 2 days)^1,7^ of enoxaparin may be too brief to be effective,^10^ making early anti-Factor Xa levels a moot point. The recommended treatment duration is 7–10 days.^5^ In a recent study by Momeni et al.,^10^ one woman suffered a pulmonary embolism 10 days after surgery (VTE rate, 3.3%) from an undetected deep venous thrombosis (DVT), despite enoxaparin injections during her hospitalization. A screening sonogram might have prevented this complication. In a study using ultrasound surveillance (1000 patients, VTE rate 0.9%),^3^ a large proximal DVT (pulmonary embolism risk, 50%^3^) was detected the day after surgery in one outpatient who received an inferior vena cava filter the same day, was anticoagulated, and developed no pulmonary embolism.

The authors dismiss screening methods (ie, ultrasound),^1^ as not recommended by “current”

2012 guidelines.^11^ This Grade 2C recommendation, based on the authors’ opinion, was made for general surgery patients, not plastic surgery patients. A Grade 2C recommendation is the weakest level of evidence, susceptible to change with higher-quality research.^12^ That new evidence is available.^3^ Ultrasound surveillance, unlike anti-Factor Xa levels, directly detects DVTs, and is highly practical and available for outpatients and inpatients.^3^ Risk prediction models, chemoprophylaxis, and anti-Factor Xa levels may be eliminated.^3^ Importantly, an increased bleeding risk is avoided. Not surprisingly, surveyed patients prefer ultrasound screening to enoxaparin injections.^3^

## References

[1] Pannucci CJ Fleming KI Bertolaccini C Prazak AM Stoddard GJ Momeni A Double blind randomized clinical trial to examine the pharmacokinetic and clinical impacts of fixed dose versus weight-based enoxaparin prophylaxis: a methodological description of the FIxed or Variable Enoxaparin (FIVE) Trial. Plast Reconstr Surg Glob Open. 2019;7:e2185. 31321183 10.1097/GOX.0000000000002185 PMC6554177

[2] Pannucci CJ Hunt MM Fleming KI Prazak AM Weight-based dosing for once-daily enoxaparin cannot provide adequate anticoagulation for venous thromboembolism prophylaxis. Plast Reconstr Surg. 2017;140:815–822. 28953735 10.1097/PRS.0000000000003692

[3] Swanson E Prospective study of Doppler ultrasound surveillance for deep venous thromboses in 1000 plastic surgery outpatients. Plast Reconstr Surg. Accepted February 2019. 10.1097/PRS.0000000000006343 31881608

[4] Lovenox (enoxaparin) Information. https://www.fda.gov/Drugs/DrugSafety/PostmarketDrugSafetyInformationforPatientsandProviders/ucm373741.htm Accessed April 20, 2019.

[5] Lovenox (enoxaparin sodium injection). Prescribing information. http://products.sanofi.us/lovenox/lovenox.pdf. Accessed April 20, 2019.

[6] Pannucci CJ Fleming KI Agarwal J Rockwell WB Prazak AM Momeni A The impact of once- versus twice-daily enoxaparin prophylaxis on risk for venous thromboembolism and clinically relevant bleeding. Plast Reconstr Surg. 2018;142:239–249. 29649055 10.1097/PRS.0000000000004517

[7] Minimization of bleeding related adverse drug events in plastic and reconstructive surgery. https://clinicaltrials.gov/ct2/show/NCT03212365. Accessed April 20, 2019.

[8] McCarthy CM Collins ED Pusic AL Where do we find the best evidence? Plast Reconstr Surg. 2008;122:1942–1947; discussion 1948. 19050548 10.1097/PRS.0b013e31818d2098

[9] Swanson E Venous thromboembolism risk stratification and chemoprophylaxis: a meta-analysis finds no benefit, more risk. Plast Reconstr Surg Glob Open 2017;5:e1356. 28740771 10.1097/GOX.0000000000001356 PMC5505832

[10] Momeni A Sorice SC Li AY Nguyen DH Pannucci C Breast reconstruction with free abdominal flaps is associated with persistent lower extremity venous stasis. Plast Reconstr Surg. 2019;143:1144e–1150e. 10.1097/PRS.0000000000005613 30907811

[11] Gould MK Garcia DA Wren SM Prevention of VTE in nonorthopedic surgical patients. Antithrombotic Therapy and Prevention of Thrombosis, 9th ed: American College of Chest Physicians Evidence-Based Clinical Practice Guidelines. Chest 2012;141(2)(Suppl):e227S–e277S. 22315263 10.1378/chest.11-2297 PMC3278061

[12] Guyatt GH Norris SL Schulman S Methodology for the development of antithrombotic therapy and prevention of thrombosis guidelines. Antithrombotic Therapy and Prevention of Thrombosis, 9th ed: American College of Chest Physicians Evidence Based Clinical Practice Guidelines. Chest 2012;141(2)(Suppl):53S–70S. 22315256 10.1378/chest.11-2288 PMC3278053

